# Spatial Pattern of Host Tree Size, Rather than of Host Tree Itself, Affects the Infection Likelihood of a Fungal Stem Disease

**DOI:** 10.3390/biology13080616

**Published:** 2024-08-14

**Authors:** Yanli Shi, Xinbo Gao, Yunxiao Jiang, Junsheng Zhang, Feng-Hui Qi, Tian-Zhong Jing

**Affiliations:** 1 School of Forestry, Northeast Forestry University, Harbin 150040, China; 2 Inner Mongolia Forest Industrial Group, Yakeshi 022150, China; 3 College of Life Sciences, Northeast Forestry University, Harbin 150040, China

**Keywords:** *Inonotus hispidus*, *Fraxinus mandshurica*, spatial point pattern analysis, generalized linear model, meta-analysis

## Abstract

**Simple Summary:**

Spatial patterns are characteristics of spatial processes, although they do not always match each other exactly. An ordinary way to explore the spatial progresses driving these spatial patterns is spatial pattern analysis, which has been widely employed in ecology studies but not in the studies of forest diseases. For diseased forest trees, the spatial pattern is a complex of the spatial pattern of forest trees and the disease itself. So, it is necessary to explore whether an antecedent pattern of host/nonhost trees affects the spatial pattern of a forest disease. Another subject that is neglected is the effect size of an antecedent pattern. In this study, taking a stem fungal disease caused by *Inonotus hispidus* as an example, we explored the two questions. Our results showed that the spatial pattern of host size affected the spatial pattern of the infection and the infection likelihood of the focal tree. Our results provide a new perspective to understand the effect of host patterns on forest disease.

**Abstract:**

The spatial pattern of diseased forest trees is a product of the spatial pattern of host trees and the disease itself. Previous studies have focused on describing the spatial pattern of diseased host trees, and it remains largely unknown whether an antecedent spatial pattern of host/nonhost trees affects the infection pattern of a disease and how large the effect sizes of the spatial pattern of host/nonhost trees and host size are. The results from trivariate random labeling showed that the antecedent pattern of the host ash tree, *Fraxinus mandshurica*, but not of nonhost tree species, impacted the infection pattern of a stem fungal disease caused by *Inonotus hispidus*. To investigate the effect size of the spatial pattern of ash trees, we employed the SADIE (Spatial Analysis by Distance IndicEs) aggregation index and clustering index as predictors in the GLMs. Globally, the spatial pattern (*v_i_* index) of ash trees did not affect the infection likelihood of the focal tree; however, the spatial pattern of DBH (diameter at breast height) of ash trees significantly affected the infection likelihood of the focal tree. We sampled a series of circular plots with different radii to investigate the spatial pattern effect of host size on the infection likelihood of the focal tree locally. The results showed that the location (patch/gap) of the DBH of the focal tree, rather than that of the focal tree itself, significantly affected its infection likelihood in most plots of the investigated sizes. A meta-analysis was employed to settle the discrepancy between plots of different sizes, which led to results consistent with those of global studies. The results from meta-regression showed that plot size had no significant effects.

## 1. Introduction

An individual host’s likelihood of acquiring an infectious disease mainly depends on the host’s location relative to sources of infection, proximity to other hosts, and the occupation of specific microhabitats that confer increased susceptibility [1]. That is, the spatial pattern of host and pathogen populations is expected to be critical in determining patterns of disease occurrence and dynamics [1]. However, there is little evidence to support this statement, since most previous studies focused on describing the spatial pattern of disease. For example, the spatial patterns of plant diseases have been studied extensively, using join-count statistics, continuous spatial autocorrelation, Mantel’s test, and Moran’s I test [1]. Recently, methods of spatial point pattern analysis (SPPA) were employed in studies of tree disease caused by *Phytophthora megakarya* [2] and mal secco [3]. In addition, using Spatial Analysis by Distance IndicEs (SADIE), strong evidence for the aggregation of fire blight was found within an orchard block [4]. Unfortunately, these studies did not couple the spatial pattern of the host to the spatial pattern of disease or the infection likelihood of the disease.

Very recently, an aggregated pattern of infected hosts was explained in part by a template of potential host distribution using a system of mistletoe and its hosts [5]. That is, the spatial pattern of diseased hosts is essentially a synthesis of the host pattern and the disease pattern itself (how the infection distributed among hosts given that an antecedent pattern of the host existed) [6]. However, there are no similar studies on the disease caused by fungi which are the main pathogens of tree disease. Using a study system of a fungal stem disease, shaggy bracket (caused by *Inonotus hispidus*), and its host, Manchurian ash (*Fraxinus mandshurica* Rupr.), in this study, we hypothesized that an antecedent spatial pattern of host trees would affect the spatial pattern of the infection (HP1). For forest pathogens, especially for those that mainly infect older/larger trees, there is an antecedent spatial pattern of the host tree before infection. We expected that this antecedent pattern would impact the infection pattern. Previous studies also suggested that high tree diversity is more likely to decrease than increase disease risk (reviewed in Koricheva et al. [7]). For example, a very recent study showed that species richness was negatively associated with disease-caused mortalities [8]. These results suggested that nonhost tree species play an important role in the outbreak of forest disease. However, it is largely unknown whether the spatial pattern of nonhosts affects the infection pattern. In this study, we hypothesized that an antecedent spatial pattern of nonhost trees would affect the infection pattern (HP2). For some pathogens, the spread rate and extent are thought to be partially controlled by host size [9]. In addition to host size itself, in this study, we hypothesized that there is an association between its spatial pattern and the infection pattern (HP3). We employed statistics of trivariate random labeling and bivariate mark correlation functions to investigate the effect of host tree patterns, nonhost tree patterns, and host size patterns. 

Then, we further investigated the effect size of the spatial patterns of host trees, host size, and nonhost trees, respectively. We hypothesized that, in this study, the aggregation of host trees/host size/nonhost trees in a circular plot would affect the disease risk of the focal host tree, and the locations (in a patch/gap) of the focal host tree/host size would also affect its disease risk. We expected that an aggregated pattern would promote the infection probability of a focal tree (HP4) and that a focal tree in a patch has a higher infection probability than a focal tree in a gap (HP5) since aggregation is helpful for disease transmission. We set up generalized linear models (GLMs) to test hypotheses HP4 and HP5, respectively. We also assumed that the results from multi-size plots would be more robust than those from single-size plots. To settle the discrepancy among plot size scales, a meta-analysis was performed. 

## 2. Materials and Methods

### 2.1. Study System

*Inonotus hispidus* (Bull. Fr.) Karst., commonly known as shaggy bracket, decays a broad range of angiosperms throughout the Northern Hemisphere [10,11]. In Europe, it is the most frequent cause of decay in ash (*Fraxinus excelsior* L.) and is also found on other broadleaved species including apple (*Malus* spp.), London plane (*Platanus × hispanica* Muenchh.), walnut (*Juglans regia* L.), and, more rarely, elm (*Ulmus* spp.), sycamore (*Acer pseudoplatanus* L.), lime (*Tilia* spp.), and Japanese pagoda tree (*Sophora japonica* L.) [10]. Conifers such as *Abies* app. can also be infected [12].

In the United States, *I. hispidus* is reported to occur most frequently on willow oak and water oak but also infects Nuttall’s oak (*Quercus nuttallii* Palmer), white oak (*Quercus alba* L.), and hickory (*Carya* spp.) [13]. In China, it is found most commonly on Manchurian ash (*Fraxinus mandshurica* Rupr.) and rarely on lime (*Tilia* spp.), poplar (*Populus* spp.), and Japanese pagoda tree [14].

This fungus is a primary parasite in most of its hosts. Airborne basidiospores transported by wind or rain are able to cause mortality of entire plant stands [12]. Hardwood stands in the delta region of Mississippi are often infested with *I. hispidus* [13]. It infected 54% of *Fraxinus*, 30% of *Platanus*, and 20% of *Populus* trees planted along avenues in urban areas in Tlemcen, the western region of Algeria [15], and 16% of walnut trees in Uzbekistan [16]. Although it has been frequently classified as a heart-rotting fungus, *I. hispidus* is capable of attacking young sapwood [10]. It mainly infects living hosts and usually forms a large, spindle-shaped canker at the site of an old branch stub 12 to 15 feet or more up the bole of the infected tree, commonly known as hispidus canker [13]. The wood behind the canker becomes soft and delignified, producing a soft-rot-like decay pattern under natural conditions [11].

**Figure 1:**
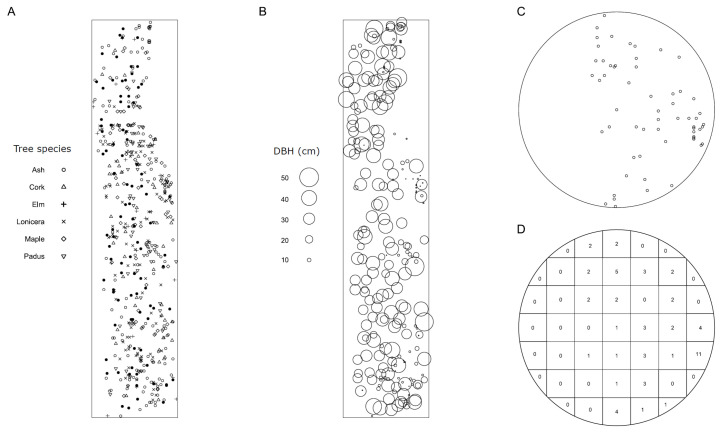
Study site and an example of a circular plot. Surveys were conducted at Harbin Experimental Base of Northeast Forestry University. (**A**) Species composition of the site, where solid circles represent infected ash trees; (**B**) DBH of ash trees; (**C**) an example of circular plot; and (**D**) quadrat count of C for SADIE analysis.

The survey was conducted at the Harbin Experimental Base attached to Northeast Forest University (HEB-NEFU) (126.62° E, 45.72° N). The HEB-NEFU includes an artificial stand with good natural regeneration and a continuous distribution on the planar stand with homogeneous environmental conditions at the base. Therefore, patterns of tree positions can be considered samples of planar homogeneous (or stationary) point fields [17]. The investigation window was 53.5 × 247.5 m, composed of 241 ashes (*F. mandshurica*), 49 corks (*Phellodendron amurense*), 21 elms (*Ulmus pumila*), 112 *Lonicera japonica*, 55 maples (*Acer negundo*), 69 *Padus racemosa*, and other trees with fewer than 10 individuals, which were not considered in this study (Figure 1A). We recorded the position of each tree and the infection status (yes or no, by whether there were sporocarps on the bole) and diameter at breast height (DBH) (Figure 1B) of each ash tree. The count of infected ash trees was 93, accounting for 38.59 ± 3.13% (mean ± se). The mean DBHs for infected and uninfected ash trees were 30.62 ± 12.80 cm (mean ± sd) and 15.79 ± 7.26 cm, respectively.

### 2.2. SADIE Analysis

The corner base of SADIE is the distance to regularity (*D*) [18], which is the minimum value of the total distance that individuals must move to make all units have the same number of individuals. The data used in SADIE are usually counts within quadrats. Individuals in the units (donors) with counts greater than the average of all units should move to units (receptors) with counts less than the average to obtain regularity. 

SADIE employs a transportation algorithm [19] to calculate *D* and a randomization test to measure the significance of aggregation. If *n* permuted values of *D* (*D*_rand_) are greater than the observed *D* out of *m* permutations, the probability for aggregation will be *P*_a_ = *n*/*m* given the null hypothesis of complete spatial randomness (CSR) is true. Letting *E*_a_ be the mean of *D*_rand_, *I*_a_ = *D*/*E*_a_ is calculated by SADIE as the index of aggregation to measure spatial aggregation. A value of *I*_a_ = 1 indicates a random spatial pattern (dummy variable name: *Ia.CatNonsig*), *I*_a_ > 1 aggregated (dummy variable name: *Ia.CatAggregation*), and *I*_a_ < 1 uniform (dummy variable name: *Ia.CatUniform*). *P*_a_ < 0.025 or > 0.975 indicates significant aggregation or regularity in the observed data, respectively [20].

SADIE uses clustering indices (*v_i_*, *v_j_*) to determine patches and gaps. Let donor *i* locate on (*x_i_*, *y_i_*), and it has *c_i_* individuals to be moved to its *r_i_* receptors to obtain regularity. And letting *d_i_* be the average distance of outflow for donor *i*, *C_i_* be the average distance of outflow for *c_i_* after *m* permutations, *Y_i_* be the average moved distance of unit (*x_i_*,*y_i_*) after *m* permutations, and *M* be the average distance of outflow for all counts after *m* permutations, then clustering index *v_i_* = *d_i_M*/(*C_i_Y_i_*). A value of *v_i_* > 1.5 indicates that unit *i* is located in a patch (dummy variable name: *vi*.*CatPatch*), *v_i_* < −1.5 indicates a gap between patches (dummy variable name: *vi*.*CatGap*), and other values indicate a random pattern (dummy variable name: *vi*.*CatRandom*). Then, a map named the red–blue plot can be used to visualize patches and gaps.

A circular plot (Figure 1C) was divided into quadrats (Figure 1D) according to the following rules to guarantee that the focal tree on the origin was in the center quadrat. Let *N* be the truncated square root of the total number of trees within a plot. The plot was divided into 9 quadrats if *N*−1 was no more than 3; otherwise, the plot was divided into (*N*−1) × (*N*−1) (where *N* is an even number) or (*N*−2) × (*N*−2) (where *N* is an odd number) quadrats.

### 2.3. Random Labeling and Trivariate Random Labeling

The mark connection function was used to test the hypothesis of whether the infected ash trees are shuffled randomly with uninfected ash trees. Mark connection functions are closely related to pair-correlation functions and product densities, which are defined as

(1) $$p_{l,m}(r)=p_{l}p_{m}g_{l,m}(r)/g_{l+m,l+m}(r)$$

where *p_l_* and *p_m_* are the proportions of type *l* and *m* points in the pattern, respectively. The pair correlation function of a stationary point process is

(2) $$g(r)=K^{\prime }(r)/\left(2\pi r\right)$$

where *K*′(*r*) is the derivative of Ripley’s *K* function. The cross-type (type *l* to type *m*) of *g*(*r*) for a multitype point pattern is defined as

(3) $$g_{l,m}(r)=K_{l,m}^{\prime }(r)/\left(2\pi r\right)$$

where *K_l_*_,*m*_′(*r*) is the derivative of the cross-type of the *K* function.

The expectation of the mark connection function under random labeling yields *p_lm_*(*r*) = *p_l_p_m_*. Values larger than this, *p_lm_*(*r*) > *p_l_p_m_*, indicate a positive association between the two types, while smaller values indicate a negative association.

To test the influence of conspecific host trees on infection, an analogous test statistic was employed [21]:

(4) $$p_{l,l+m}(r)=p_{l}g_{l,l+m}(r)/g_{l+m,l+m}(r)$$

To test the influence of heterospecific nonhost trees on infection, trivariate random labeling, another analogous test statistic, was employed [21]:

(5) $$p_{l,h}(r)=p_{l}g_{l,h}(r)/g_{l+m,h}(r)$$

Trivariate random labeling explores the effect of an antecedent focal pattern *h* on the process that distributes a qualitative mark (type *l* and type *m*) over a second pattern [22]. Under random labeling, the expectations of the two analogous test statistics are *p_l_*. Larger statistics (>*p_l_*) suggest negative interactions exerted by hetero- or conspecific trees, and smaller statistics (<*p_l_*) suggest positive interactions.

Simulation envelopes arising from random infection (i.e., random permutation of labels *l* and *m*) were used to compare these summary statistics.

### 2.4. Spatial Point Pattern Test

Univariate Ripley’s *K* functions [23], *K*(*r*), were used to describe the spatial patterns of ash/nonhost trees. The cross-type *K* function of a stationary multitype point process X is defined so that *λ_l_K_l,m_*(*r*) equals the expected number of additional random points of type *m* within a distance *r* of a typical point of type l in the process X. Here, *λ_m_* is the intensity of the type *m* points. The null hypothesis for the cross-type *K_lm_*(*r*) function in this study is that the locations of the type *l* and *m* points result from two a priori independent spatial point processes [24]. To generate envelope simulations, 2499 simulations of complete spatial randomness (CSR) with the same intensity as the patterns of ash and nonhosts were generated for *K*(*r*) and *K_l,m_*(*r*), respectively.

### 2.5. Testing the Effect of DBH on Infection Patterns

Bivariate mark-correlation functions were employed to test the hypothesis that DBH shows a spatial correlation with infection [25]. In this case, we have a spatial point pattern of ash trees with one qualitative mark (i.e., infected and uninfected) and one quantitative mark (i.e., DBH). The bivariate mark-correlation functions relate the size of the focal label (infected) to the size of ash trees of all other labels (uninfected) that are located at distance *r* away from the focal ash tree [22]. The estimator of the nonnormalized mark-correlation functions is

(6) $${\hat{k}}_{lm}(r)=\frac{\sum_{i=1}^{n}\sum_{j=1}^{n,\neq }t(m_{il},m_{jm})\times I_{lm}(x_{i},x_{j})\times h(\left\|x_{i}-x_{j}\right\|-r)\times w_{i,j}}{\sum_{i=1}^{n}\sum_{j=1}^{n,\neq }I_{lm}(x_{i},x_{j})\times h(\left\|x_{i}-x_{j}\right\|-r)\times w_{i,j}}$$

where the indicator function *I_lm_*(*x_i_*, *x_j_*) equals 1 if point *i* is a type *l* point and point *j* is a type *m* point; otherwise, it is 0. *t*(*m_il_*_,_ *m_jm_*) is the test function that uses the mark *m_i_* of point *l* and the mark *m_j_* of point *m*; *h*() is the kernel function that defines which points are located approximately at distance *r*; and *w_ij_* is the edge correction. Here, we employed four mark-correlation functions [22]:

(7) $$\begin{array}{l} k_{m}(r):t_{1}(m_{il},m_{jm})=m_{il}\\ k_{mm}(r):t_{2}(m_{il},m_{jm})=m_{il}m_{jm}\\ \gamma_{mm}(r):t_{3}(m_{il},m_{jm})={\left(m_{il}-m_{jm}\right)}^{2}/2\\ I_{mm}(r):t_{4}(m_{il},m_{jm})=(m_{il}-\mu_{l+m})(m_{jm}-\mu_{l+m}) \end{array}$$

The test function *t*_1_ yields the mean mark of type *l* points (e.g., infected), which are distance *r* away from a type *m* point (e.g., uninfected). The test function *t*_2_ yields a bivariate mark-correlation function *k_m_*_1*m*2_(*r*), which returns the mean product of the marks of all pairs of type *l* and *m* points that are a distance *r* apart. A value of *k_mm_*(*r*) larger than 1 indicates that the mean mark product of the typical point and its nearest neighbor is above the average. By definition, *k_mm_*(*r*) = 1 if the marks are independent and *k_mm_*(*r*) <1 if there is mutual inhibition. The mark variogram function *t_3_* yields the mean squared difference in the marks of all pairs of type *l* and type *m* points, which are a distance *r* apart [26]. The bivariate-mark variogram measures whether the sizes of neighboring infected and uninfected ash trees are more similar (low values) or dissimilar (high values) than the sizes of a pair of trees taken at random [22]. The correlation function t_4_ yields a Moran’s I-type summary statistic, *I_m_*_1*m*2_(*r*), which characterizes the covariance, a second basic property of the spatial correlation structure between the marks of all pairs of type *l* and type *m* points separated by distance *r* [22].

Envelope simulations randomized the mark of DBH 2499 times.

### 2.6. Generalized Linear Models (GLMs) and Meta-Analysis

Circular instead of rectangular plots were employed in this study. To investigate the effect of plot size, the plot radii were assigned from 1 m to 25 m with a step of 1 m, which yielded 22 (*r* = 25 m) to 241 (*r* = 1 m) plots. The centers of circles were ash trees (focal trees) with distances of no less than the radii from the nearest border of the investigation window, ensuring that all plots were complete circles and that at least one ash tree was included in a plot. GLMs were established with logit link functions and a common binary response variable, i.e., infection status of the focal tree (“yes” or “no”). The explanatory variables were the SADIE aggregation index and clustering index, which were grouped into categories. Models were set up for each plot radius. Subsequently, the coefficients and their SEs from models for 6–25 m plots were subjected to meta-analysis to obtain the pooled effect size and test the effect of plot size (radius). Higgins and Thompson’s *I*^2^ was used to determine between-study heterogeneity, and the inverse variance method was used to pool effect size under a random-effects model. Meta-regression analysis was performed using a mixed-effects model.

### 2.7. Statistics and Software

Statistics were performed using R (4.0) packages [27]. The packages idar [28], stats [27], meta [29], and spatstat [30] were employed for tree counting, GLM fitting, meta-analysis, and spatial point pattern analysis (SPPA), respectively. The R package epiphy [31] was used to calculate indices of aggregation and clustering. We also developed an R package, markSPPA, for bivariate/trivariate marked spatial point pattern analysis (https://github.com/Gitjtz/markSPPA/tree/master, accessed on 22 November 2023).

## 3. Results

### 3.1. Correlation between Infection and Antecedent Spatial Patterns of Hosts/Nonhosts

**Figure 2:**
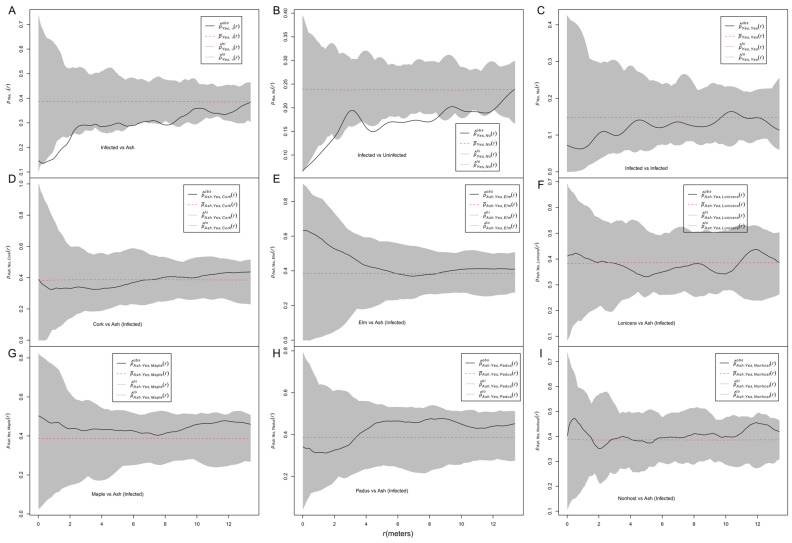
Random labeling and trivariate random labeling analysis. (**A**) Conspecific trivariate random labeling *p*_1,1+2_(*r*) exploring the effect of the spatial pattern of conspecific plants on the infection pattern; (**B**) the test statistic *p*_12_(*r*) exploring attraction versus segregation of infected and non-infected hosts; (**C**) the test statistic *p*_11_(*r*) exploring the aggregation of infected hosts; (**D**–**I**) heterospecific trivariate random labeling *p*_1*,f*_(*r*) exploring the effect of the pattern of nonhost plants on the infection pattern. The categories “yes” and “no” represent infected and uninfected ash trees, respectively. The dot in “*p*_yes,•_” in A represents all ash trees (“yes” + “no”, i.e., both infected and uninfected ash trees). “Nonhost” in I represents a pool of all nonhost tree species (Cork+Elm+Lonicera+Maple+Padus). If the test statistic is below the simulation envelopes, the infection is lower than expected, and if it is above the simulation envelopes, the infection is higher than expected.

Conspecific trivariate analysis with random labeling between infected and all ash trees showed that *p*_yes,•_, was smaller than expected at small distances, indicating that the presence of ash trees of a given pattern influenced the spatial pattern of infection (Figure 2A). The analysis with the mark connection function showed that the *p_yes,no_* curve was smaller than expected at small distances, indicating a negative association between marks of “infected” and “uninfected” over all ash trees (Figure 2B). The *p_yes,yes_* curve was within the envelopes, indicating a random labeling of “infected” marks. The *p_ash,heterospecies_* curves were neither above nor below the envelopes, indicating that the presence of nonhost trees of a given pattern did not influence the spatial pattern of infection (Figure 2D–I).

### 3.2. Spatial Patterns of Ash Trees and Their Sizes

**Figure 3:**
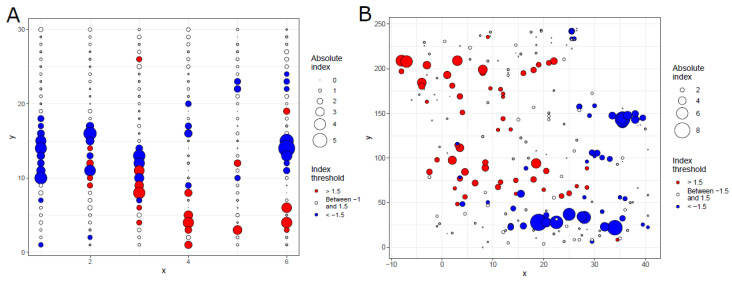
Global SADIE analysis of ash trees and DBH of ash trees. (**A**) Red–blue plot of the spatial pattern of ash trees; (**B**) red–blue plot of the spatial pattern of DBHs (diameters at breast height) of ash trees. Circles represent quadrats in panel (**A**), whereas they represent ash trees in panel (**B**), and axes are their spatial coordinates. The size of the circles represents the absolute values of clustering indices *v_i_*, while the color represents the spatial pattern (red, patch, *v_i_* > 1.5; blue, gap, *v_i_* < −1.5; white, random, *v_i_* between −1.5 and 1.5).

Globally, the aggregation indices (*I_a_*) were 1.3786 (*P_a_* = 0.1106) and 1.8380 (*P_a_* = 0.0250) for ash trees and their DBH, respectively, indicating that either of them is aggregated. The patterns of ash trees and their sizes are shown in Figure 3.

**Figure 4:**
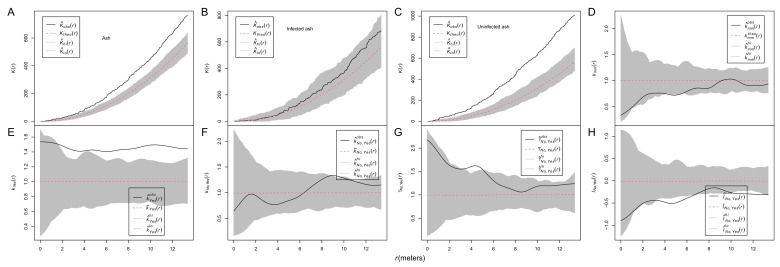
Spatial pattern of ash trees and their DBH. Ripley’s *K* function for an unmarked point pattern (**A**–**C**); if the test statistics are above the simulation envelopes, the unmarked ash trees are aggregated; conversely, they are inhibited. The mark correlation function *k_mm_*(*r*) assesses the spatial pattern of the DBH of ash trees (**D**). Bivariate mark-correlation function analysis for one qualitative mark (infection (“yes”) and no infection (“no”)) with one quantitative mark (DBH) (**E**–**H**): r-mark-correlation function analysis for mark “yes” (**E**), r-mark-correlation functions (**F**), mark variogram functions (**G**), and Moran’s I mark-correlation function (**H**). A value of 1 of the test statistic *k_mm_*(*r*) suggests a lack of correlation. If the test statistic *k_yes_*(*r*) is above/below the simulation envelopes, large/small infected ash trees are aggregated, with ash trees at distance *r*. If the test statistic *k_yes,no_*(*r*) is above/below the simulation envelopes, large/small infected ash trees are aggregated at distance *r* with large/small uninfected ash trees, given the overall sizes of infected and uninfected trees. If the test statistic γ_yes,no_(*r*) is above/below the simulation envelopes, infected ash trees are aggregated at distance *r* with uninfected ash trees of different/similar sizes. If the test statistic *I_yes,no_*(*r*) is above/below the simulation envelopes, there is a positive/negative spatial correlation between the DBHs of infected and uninfected ash trees at distance *r*.

The results from Ripley’s *K* functions showed that ash trees were aggregated (unmarked *K*(*r*), Figure 4A), infected ash trees were randomly distributed (Figure 4B), and uninfected ash trees were aggregated (Figure 4C). The results from the mark correction function *k_mm_*(*r*) for marked points showed that the DBH of ash trees was neither aggregated nor inhibited (Figure 4D).

A set of mark correlation functions was employed to investigate the spatial patterns of ash sizes. The r-mark-correlation function *t*_1_ (*k_m_*_1_(*r*)) indicated that the size of infected ash trees located at distances *r >* 2 m from uninfected ash trees was significantly larger than expected (Figure 4E). The test function *t*_2_ (*k_m_*_1*m*2_(*r*)) showed that the size of infected and uninfected ash trees located at distance *r* was not significantly smaller/larger than expected (Figure 4F). The mark variogram function *t*_3_ showed that infected and uninfected ash trees had significantly dissimilar sizes at distances of 4–5.5 m apart, and there was a trend that nearby infected and uninfected trees tended to have more similar sizes with distance (Figure 4G). Moran’s correlation function *t*_4_ was below zero, indicating that big/small infected ash trees aggregated with small/big uninfected ash trees at most investigated distances, given the group mean of infected trees or uninfected trees, respectively (Figure 4H).

### 3.3. Spatial Pattern of Nonhost Species

**Figure 5:**
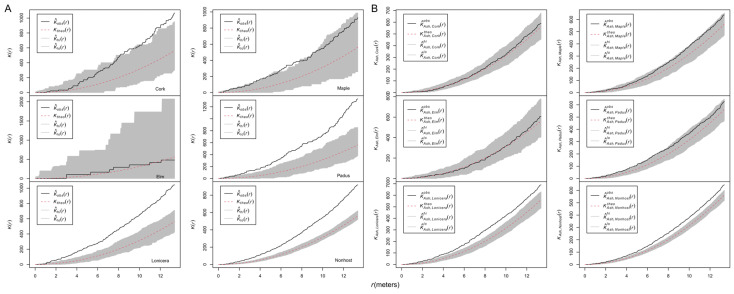
Spatial patterns of nonhost species. (**A**) Ripley’s *K* functions for the unmarked point patterns of nonhost species; (**B**) cross-type K function analysis for ash and nonhost species. Curves above/below the simulation envelopes suggest spatial clustering/regularity.

The *K*(*r*) analyses were performed to explore the spatial pattern of nonhosts (Figure 5A). The *K*(*r*) curve of cork was above the envelopes at larger distances (*r* > 11 m), indicating aggregations at these distances. The aggregation of maple was only found at 6–8 m. *Lonicera, Padus,* and the pooled nonhosts were aggregated at almost all investigated distances. The *K*(*r*) curve of elm was within the envelope, indicating that elm was randomly distributed. The cross-type *K*_1,2_(*r*) analyses were employed to explore the independence between ash and nonhost trees (Figure 5B). Ash trees were independent from all nonhost trees except for *Lonicera* and the nonhost pool, which showed attraction patterns.

### 3.4. Spatial Pattern Effect of Ash Trees, Their DBHs, and Nonhost Trees on Infection Likelihood

Globally, 18 trees were located in gaps, 60 trees were in patches, and others were distributed randomly (Supplementary Data S1); 54 trees were located in gaps of the DBH, 54 trees were in patches of the DBH, and others were distributed randomly in terms of the DBH pattern (Supplementary Data S2). The spatial pattern (*vi* index) of ash trees did not affect the infection likelihood of the focal tree; however, the spatial pattern of the DBH of ash trees significantly affected the infection likelihood of the focal tree (Table 1). Compared to those located in the gaps of the DBH, the infection odds of the trees located in patches and distributed randomly increased 23.28 (*e*^3.1474^) times and 5.90 (*e*^1.7756^) times, respectively.

Locally, only a few plots had aggregated or inhibited ash trees (Supplementary Data S3), and only a few plots had inhibited the DBH of ash trees (Supplementary Data S4). Smaller plots (radius < 20 m) had a more randomly distributed DBH of ash trees while larger plots (radius > 20 m) had a more aggregated DBH of ash trees (Supplementary Data S4). None of the focal trees were located in the gaps of ash trees, a few of the focal trees were located in patches of ash trees, and most of them were distributed randomly (Supplementary Data S5). A few of the focal trees were located in patches or gaps of the DBH of ash trees, and most of them were distributed randomly (Supplementary Data S6). Neither the aggregation (*I_a_* index) of ash trees in a plot nor the aggregation (*I_a_* index) of the DBH of ash trees in a plot significantly affected the infection likelihood of the focal ash tree (Tables S1 and S2). The location (gap/random/patch) of a focal tree did not significantly affect its infection likelihood in all plots of the investigated sizes (Table S3); however, the location of the DBH of a focal tree significantly affected its infection likelihood in most plots of the investigated sizes (Table 1 and Table S4). Nonhost trees were distributed randomly in most plots, and only a few were aggregated or inhibited (Supplementary Data S7). The results from GLM models also showed that the aggregation (*I_a_* index) of pooled nonhost tree species in a plot did not significantly affect the infection likelihood of the focal ash tree (Table S5).

The results from the meta-analyses showed that the coefficient of the explanatory variables *vi*.*CatRandom* and *vi*.*CatPatch* in the model was not heterogeneous (*I*^2^ = 0) (Table 2). The pooled coefficients for *vi*.*CatRandom* and *vi*.*CatPatch* were 1.6772 and 1.9380, respectively (Table 2), indicating that, compared to those located in gaps of the DBH, the infection odds of the trees located in patches and distributed randomly increased 6.94 (*e*^1.9380^) times and 5.35 (*e*^1.6772^) times, respectively. Meta-regression results showed that the moderator variable plot radius did not have a significant effect (Table 1, *p* > 0.05).

The effect of DBH itself was also investigated using a GLM. The results showed that DBH had a significant positive coefficient of 0.1162 (se = 0.0160, z = 7.2803, *p* = 3.33 × 10^−13^).

## 4. Discussion

The spatial pattern of a forest disease is determined by multiple factors and is scale-dependent. Spatial patterns at large scales are often induced by the Moran effect while being induced by autocorrelation at fine scales [32]. In this study, we did not pay any attention to the Moran effect but aimed to explore the effect of host/nonhost spatial pattern on the infection of a forest disease caused by *I. hispidus*, although the host species descriptors such as host abundance and the composition of host species assemblages have been shown to play a role in forest disease epidemics [33]. Most previous studies often study this issue using random field models at large scales. For example, a study across six European countries showed that the susceptibility of forests to disease appears to depend on the forest site [34]. Another study across 32 countries showed that the geographic distance between locations is one of the major drivers of dissimilarities of tree-associated fungal communities [35]. Compared to the previous studies, our results provide a new perspective to understand the effect of host patterns on forest disease.

Using an approach of SPPA, the spatial pattern of infected ash trees was determined as randomness at all investigated distances (Figure 4B). The spatial pattern of infected ash trees is a product of the spatial pattern of the host tree and the spatial pattern of the infection itself [6], since fungi related to plants are often host-dependent [36]. We investigated whether an antecedent spatial pattern of host trees affects the infection pattern using trivariate random labeling, and the results showed that the antecedent spatial pattern of host ash trees affected the infection of the fungal stem disease (Figure 2A). A similar result was also reported in the system of the mistletoe *Tristerix corymbosus* and its hosts [5]. We also investigated the spatial patterns of host trees and the infection, which is also helpful for the precision management of this disease. Our results suggested that the aggregated spatial pattern of host trees and the negative association between the “infected” and “uninfected” marks synthesized a random spatial pattern of infected ash trees (Figure 4B), which lacks other supporting evidence. 

However, an aggregated pattern of infected hosts was found in the system of mistletoe *T. corymbosus* and its hosts, which was caused in part by the template of the potential host distribution but was subsequently modulated by the activity of the disperser [5], a small nocturnal and arboreal marsupial [37], whereas mechanical injury or bark beetles and stem borer insects are the main sources of fungal infection [38]. In our study, no stem borers of ash were observed. Thus, mechanical injury is the main cause of the infection for this disease. The difference also probably lies in the fact that host size (DBH) significantly affected the infection pattern in our study system but not in the system of mistletoe *T. corymbosus* and its hosts [5]. Since the infection was host-size-dependent, the distribution of the DBH perhaps also determined the distribution of marks “infected” and “uninfected” among ash trees. Given the aggregated distribution of ash trees (Figure 4A), the DBHs were randomly assigned among ash trees (Figure 4D), which is probably the main reason for the segregated, rather than aggregated, pattern of the marks of “infected” and “uninfected” (Figure 2B). Since the pattern of DBH is an antecedent pattern of the infection, the negative spatial correlation between the DBHs and the infection (Figure 4H) can be interpreted as the effect of the DBH pattern on the infection pattern. As an artificial stand, the DBHs of ash trees should be homogeneous. The variation in DBH was mainly generated by the natural regeneration of ash trees. Therefore, it seems that the natural regeneration of the host affected the spatial pattern of the forest disease, which needs further study.

In contrast to those of host trees, antecedent spatial patterns of nonhost trees did not significantly affect the spatial pattern of infection, from the point of view of either a single species (Figure 2D–H) or a pool of all nonhost species (Figure 2I), although the trees of these species except for elm were aggregated (Figure 5A), and both *Lonicera* and nonhost pool showed an attraction to ash trees (Figure 5B). Our results also showed that the spatial pattern of neighboring nonhost trees did not affect the infection likelihood of a focal host tree (Table S5). These results provide applied significance that the spatial patterns of nonhost trees are not important at the investigated distances in a silviculture of mixed stands, from the perspective of the management of this disease. In this study, we employed a series of circular sub-plots centered on a focal tree to generate a diverse spatial pattern of host/nonhost trees. Compared to generating spatial patterns by artificial experiments, for example, the BEF-China experiment [39], our approach is cost-saving. However, our approach can only utilize naturally growing species and cannot test non-existing species combinations. Furthermore, our approach can only measure the patterns as aggregation and cannot provide a clear pattern map as artificial experiments, which is more operational for management practices.

We further investigated the effect size of the spatial pattern of host trees. Globally, the location (gap/random/patch) of a host tree did not significantly affect its infection likelihood (Table 1). Locally, neither the spatial pattern (aggregation/inhibition) of host trees in a circular plot nor the location of a focal tree significantly affected the infection likelihood of the focal tree in a circular plot (Tables S1 and S3). Since plant–plant interactions between conspecifics have been shown to modulate the susceptibility to pathogens [40], the spatial pattern of the host would affect the infection likelihood. Therefore, our results were unexpected. The reason probably lies in the fact that we did not obtain enough variation in predictors. Globally, most trees were located neither in gaps nor patches (Supplementary Data S1); locally, very few plots had an inhibited pattern of host trees, very few plots had an aggregated pattern of host trees (Supplementary Data S3), none of the focal trees were located in gaps, and almost none of the trees were located in patches (Supplementary Data S5).

However, our results showed that the spatial pattern of host size (DBH) significantly affected the infection likelihood of focal trees (Table 1). Host heterogeneity has been demonstrated to affect the infection risk of cedar trees during epidemics [41]. Our results showed that the infection odds increased 1.12 times (*e*^0.1162^) with a one-unit increase in DBH, consistent with previous studies showing that most fungal pathogens causing stem disease often attack older/larger trees through mechanical injuries [38]. In this study, however, we focused on whether the spatial patterns of size (DBH) of ash trees affect the infection likelihood. The results from GLMs suggested that the local spatial pattern of DBHs of host trees in a circular plot did not significantly affect the infection likelihood of the focal trees (Table S2). However, focal host trees located in patches of DBH have a higher risk of being infected by the disease than those in gaps of DBH, either at the global or local scale (Table 1). These results are probably the reasons for the pattern where infected host trees had a significantly larger DBH given the overall sizes of infected and uninfected ash trees almost at the distances of about *r* > 2 m (Figure 4E). These results also suggested that this disease was mainly transmitted among large trees, although large trees and small trees were randomly distributed at the investigated distances (Figure 4D). Furthermore, these results provide an approach to detect this disease early. Models can be set up based on the spatial pattern of the host tree or its size to predict the infection likelihood of a focal host tree.

As presented in the Introduction section, most studies employed the SADIE aggregation index to describe spatial patterns. Xu and Madden (2004) used a power-law-type model to relate *I_a_* to disease incidence [42], which extended the usage of SADIE. In this study, we used *I_a_* and *v_i_* as predictor variables to establish GLMs, which also extended the usage of SADIE. Locally, we employed a series of circular plots with different sizes to test the effect size of the spatial pattern and investigated the effect of plot size. The results from the meta-regression showed that there were no significant effects of plot size (Table 2) given the investigated plot sizes. However, the effect sizes were plot-size-dependent (Table 1 and Table S4). To settle discrepancies arising from conflicting claims of individual studies, the pooled effect size was employed in this study. Compared to the approach of selecting the best models in terms of *R*^2^ and the AIC [43], meta-analysis retains the variations among individual studies, increases the numbers of observations and the statistical power, and improves the estimates of the effect size [44]. The pooled effect size at the local scale was consistent with that at the global scale. These results suggested the effect of spatial pattern functions at both local and global scales.

## 5. Conclusions

In this study, we explored two types of questions. One was whether an antecedent spatial pattern of host trees/host size/nonhost trees affected the infection pattern of a forest disease; the other was how large the effect was. We developed an R package, *markSPPA* (https://github.com/Gitjtz/markSPPA/tree/master accessed on 11 August 2024), to employ marked spatial point pattern analysis to answer the first type of question. The results from trivariate random labeling analyses showed that the presence of ash trees of a given pattern influenced the spatial pattern of the infection. However, the presence of nonhost trees of a given pattern did not influence the spatial pattern of the infection. The results from bivariate mark-correlation functions showed that big/small infected ash trees aggregated with small/big uninfected ash trees at most investigated distances.

We employed SADIE’s aggregation index and clustering index as explanatory variables to explore the effect size of the spatial pattern of host trees/host size/nonhost trees on the infection likelihood of a focal host tree in a circular plot. The results from GLM models showed that the spatial pattern of either host trees or its size did not significantly affect the infection likelihood. The location state of the focal tree did not significantly affect its infection likelihood, but the location state of size of the focal tree significantly affected its infection likelihood.

Our results provided applied significance to the management of this disease. Spatial information of this disease is helpful for precision management, GLM models provide an approach of early detection, and the results that the spatial pattern of nonhost trees affected neither the spatial pattern of the infection nor the infection likelihood also provided useful guidance on silviculture of ash stands.

## Figures and Tables

**Table 1:** Coefficients of GLMs for determination of the effect of spatial patterns on infection likelihood.

Model	Explanatory	Estimate	Std. Error	z Value	Pr(>|z|)	Radius of Plot (m)
global *v_i_*(tree)	(Intercept)	−6.73 × 10^−15^	4.71 × 10^−1^	0	1	
	*vi.CatRandom*	−5.94 × 10^−1^	4.99 × 10^−1^	−1.189	0.234	
	*vi.CatPatch*	−2.68 × 10^−1^	5.39 × 10^−1^	−0.498	0.618	
global *v_i_*(DBH)	(Intercept)	−2.2824	0.4694	−4.862	1.16 × 10^−6^	
	*vi.CatRandom*	1.7756	0.5024	3.534	0.0004	
	*vi.CatPatch*	3.1474	0.556	5.661	1.51 × 10^−8^	
local *v_i_*(DBH)	*vi.CatRandom*	2.4980	1.0487	2.3820	0.0172	10
	*vi.CatRandom*	2.6935	1.0468	2.5729	0.0101	11
	*vi.CatRandom*	2.2678	0.7652	2.9637	0.0030	12
	*vi.CatRandom*	2.9618	1.0446	2.8353	0.0046	13
	*vi.CatPatch*	3.1781	1.2802	2.4825	0.0130	13
	*vi.CatRandom*	2.5421	1.0590	2.4005	0.0164	14
	*vi.CatPatch*	2.3026	1.0488	2.1954	0.0281	15
	*vi.CatRandom*	1.6458	0.8310	1.9804	0.0477	19
	*vi.CatRandom*	1.9363	0.7224	2.6806	0.0073	20
	*vi.CatPatch*	2.0794	0.8898	2.3371	0.0194	20
	*vi.CatRandom*	2.1893	0.8435	2.5956	0.0094	21
	*vi.CatPatch*	2.5903	1.0288	2.5179	0.0118	21
	*vi.CatRandom*	2.5745	0.8790	2.9290	0.0034	22
	*vi.CatPatch*	3.1135	1.1005	2.8292	0.0047	22
	*vi.CatRandom*	2.1972	0.8975	2.4481	0.0144	23
	*vi.CatPatch*	3.3322	1.3296	2.5062	0.0122	23
	*vi.CatRandom*	2.8214	1.1992	2.3527	0.0186	24

**Table 2:** Meta-analysis of coefficients from GLMs.

Explanatory	*K*	*I* ^2^	Pooled Effect Size					Meta-Regression				
			Pooled Beta	Lower Bound	Upper Bound	Zval	Pval	Beta	se	Zval	Pval	*R* ^2^
*vi.CatRandom*	20	0	1.6772	1.3151	2.0392	9.0804	1.08 × 10^−19^	0.0161	0.0366	0.4406	0.6595	0
*vi.CatPatch*	20	0	1.9380	1.3748	2.5013	6.7440	1.54 × 10^−11^	0.0784	0.0631	1.2415	0.2144	0

## Data Availability

Data are contained within the article and Supplementary Materials.

## Supplementary Materials

The following supporting information can be downloaded at https://www.mdpi.com/article/10.3390/biology13080616/s1. Table S1: Coefficients of SADIE aggregation index Ia of ash tree in local models; Table S2: Coefficients of SADIE aggregation index Ia of DBH in local models; Table S3: Coefficients of SADIE clustering index vi of ash tree in local models; Table S4: Coefficients of SADIE clustering index vi of DBH in local models; Table S5: Coefficients of SADIE aggregation index Ia of pooled nonhost tree species in local GLM models; Data S1: Global frequency table for vi index of ash tree; Data S2: Global frequency table for vi index of the DBH of ash tree; Data S3: Local frequency table for Ia index of ash tree; Data S4: Local frequency table for Ia index of DBH of ash tree; Data S5: Local frequency table for vi index of ash tree; Data S6: Frequency table for vi index of DBH of ash tree; Data S7: Frequency table for Ia index of pooled nonhost tree species.
