# Supplementary material for: Spatial Pattern of Host Tree Size, Rather than of Host Tree Itself, Affects the Infection Likelihood of a Fungal Stem Disease
Source: Biology (Basel). 2024 Aug 14;13(8):616. doi: 10.3390/biology13080616 (PMC11351737; doi:10.3390/biology13080616)
Supplement: Supplementary file 1 [file biology-13-00616-s001.zip › biology-3098712-supplementary.pdf]

Supplementary Information for

Spatial Pattern of Host Tree Size, Rather than of Host Tree Itself, Affects the Infection Likelihood of a Fungal Stem Dis-ease

Table of content

Table S1	2
Table S2	4
Table S3	6
Table S4	7
Table S5	9
Data S1	11
Data S2	12
Data S3	13
Data S4	17
Data S5	21
Data S6	25
Data S7	29

Table S1.

Coefficients of SADIE aggregation index Ia of ash tree in local GLM models.

Explanatory	Estimate	Std. Error	z value	Pr(>|z|)	Plot radius (m)
(Intercept)	-0.693147181	1.22474487	-0.565952	0.5714	6
Ia.CatNonsig	0.371563556	1.23281288	0.3013949	0.7631	6
Ia.CatUniform	-13.8729206	882.744225	-0.015716	0.9875	6
(Intercept)	14.56606776	882.743374	0.0165009	0.9868	7
Ia.CatNonsig	-14.94684026	882.743386	-0.016932	0.9865	7
(Intercept)	0.693147181	1.22474465	0.5659524	0.5714	8
Ia.CatNonsig	-1.085189268	1.23383216	-0.879527	0.3791	8
(Intercept)	-16.56606841	1073.10902	-0.015437	0.9877	9
Ia.CatNonsig	16.35350697	1073.10904	0.0152394	0.9878	9
(Intercept)	-1.609437912	1.09544512	-1.469209	0.1418	10
Ia.CatNonsig	1.348222413	1.10743832	1.2174244	0.2234	10
Ia.CatUniform	16.17550569	882.744055	0.0183241	0.9854	10
(Intercept)	-1.83E-14	1.41421356	-1.29E-14	1.0000	11
Ia.CatNonsig	-0.237958635	1.42361847	-0.167151	0.8673	11
Ia.CatUniform	2.26E-14	2	1.13E-14	1.0000	11
(Intercept)	-0.693147181	1.22474487	-0.565952	0.5714	12
Ia.CatNonsig	0.482582411	1.23623753	0.3903638	0.6963	12
Ia.CatUniform	-13.8729206	882.744225	-0.015716	0.9875	12
(Intercept)	-0.160930367	0.171425	-0.93878	0.3478	13
Ia.CatUniform	-14.40513741	882.743392	-0.016319	0.9870	13
(Intercept)	1.67E-14	1	1.67E-14	1.0000	14
Ia.CatNonsig	-0.138150338	1.01717626	-0.135818	0.8920	14
Ia.CatUniform	-0.693147181	1.58113866	-0.438385	0.6611	14
(Intercept)	0.405465108	0.91287093	0.4441648	0.6569	15
Ia.CatNonsig	-0.405465108	0.93369956	-0.434257	0.6641	15
Ia.CatUniform	-15.97153336	1029.12187	-0.01552	0.9876	15
(Intercept)	0.405465108	0.91287093	0.4441648	0.6569	16
Ia.CatNonsig	-0.363792412	0.93542402	-0.388906	0.6973	16
Ia.CatUniform	-15.97153336	1455.39782	-0.010974	0.9912	16
(Intercept)	1.386294361	1.11803399	1.2399394	0.2150	17
Ia.CatNonsig	-1.295322583	1.13822171	-1.138023	0.2551	17
Ia.CatUniform	-16.95236261	1029.12207	-0.016473	0.9869	17
(Intercept)	1.098612289	1.15469954	0.951427	0.3414	18
Ia.CatNonsig	-1.029619417	1.17446276	-0.876673	0.3807	18
(Intercept)	-0.693147181	1.22474487	-0.565952	0.5714	19
Ia.CatNonsig	0.798507696	1.24610504	0.6408029	0.5217	19
Ia.CatUniform	16.25921543	1455.39805	0.0111717	0.9911	19
(Intercept)	-15.56606825	1455.39753	-0.010695	0.9915	20
Ia.CatNonsig	15.77578878	1455.39755	0.0108395	0.9914	20

(Intercept)	-16.56606843	1385.37779	-0.011958	0.9905	21
Ia.CatNonsig	16.94336266	1385.37781	0.0122301	0.9902	21
(Intercept)	-1.386294361	1.11803371	-1.23994	0.2150	22
Ia.CatNonsig	2.047692843	1.15963676	1.7658054	0.0774	22
(Intercept)	-3.52E-15	1	-3.52E-15	1.0000	23
Ia.CatNonsig	0.510825624	1.05198225	0.4855839	0.6273	23
(Intercept)	-16.56606842	2399.54471	-0.006904	0.9945	24
Ia.CatNonsig	17.2079223	2399.54474	0.0071713	0.9943	24
(Intercept)	-16.56606842	2399.54472	-0.006904	0.9945	25
Ia.CatNonsig	17.2592156	2399.54476	0.0071927	0.9943	25

Table S2.

Coefficients of SADIE aggregation index Ia of DBH in local GLM models.

Explanatory	Estimate	Std. Error	z value	Pr(>|z|)	Plot radius (m)
(Intercept)	-1.299282984	0.65134	-1.99479	0.0461	6
Ia.CatNonsig	1.018380599	0.6693	1.521565	0.1281	6
Ia.CatUniform	-14.26678526	727.699	-0.01961	0.9844	6
(Intercept)	-0.693147181	0.61237	-1.1319	0.2577	7
Ia.CatNonsig	0.339215723	0.63112	0.537481	0.5909	7
Ia.CatUniform	0.287682072	1.09924	0.261709	0.7935	7
(Intercept)	-1.098612289	0.66667	-1.64792	0.0994	8
Ia.CatNonsig	0.786581187	0.68472	1.148768	0.2507	8
Ia.CatUniform	0.405465108	1.09291	0.370997	0.7106	8
(Intercept)	-0.916290732	0.83666	-1.09518	0.2734	9
Ia.CatNonsig	0.617797743	0.85161	0.725444	0.4682	9
Ia.CatUniform	2.302585093	1.39642	1.648916	0.0992	9
(Intercept)	-16.56606841	1199.77	-0.01381	0.9890	10
Ia.CatNonsig	16.28610839	1199.77	0.013574	0.9892	10
Ia.CatUniform	16.97153352	1199.77	0.014146	0.9887	10
(Intercept)	-0.693147181	0.86603	-0.80038	0.4235	11
Ia.CatNonsig	0.448693843	0.88172	0.508884	0.6108	11
Ia.CatUniform	16.25921543	1029.12	0.015799	0.9874	11
(Intercept)	-1.945910149	1.06904	-1.82024	0.0687	12
Ia.CatNonsig	1.768979441	1.08282	1.633684	0.1023	12
Ia.CatUniform	2.63905733	1.62568	1.623352	0.1045	12
(Intercept)	-0.287682066	0.76375	-0.37667	0.7064	13
Ia.CatNonsig	0.119346752	0.78362	0.152302	0.8789	13
(Intercept)	0.33647222	0.58552	0.574657	0.5655	14
Ia.CatNonsig	-0.535323078	0.61581	-0.8693	0.3847	14
(Intercept)	0.510825624	0.5164	0.98921	0.3226	15
Ia.CatNonsig	-0.595983432	0.55614	-1.07163	0.2839	15
Ia.CatUniform	-16.07689387	1455.4	-0.01105	0.9912	15
(Intercept)	0.200670695	0.44946	0.446466	0.6553	16
Ia.CatNonsig	-0.200670695	0.50201	-0.39973	0.6894	16
Ia.CatUniform	-0.200670695	1.48392	-0.13523	0.8924	16
(Intercept)	0.318453719	0.46465	0.685368	0.4931	17
Ia.CatNonsig	-0.265809986	0.51823	-0.51292	0.6080	17
(Intercept)	0.405465108	0.45644	0.88833	0.3744	18
Ia.CatNonsig	-0.348306694	0.51529	-0.67594	0.4991	18
Ia.CatUniform	-15.97153336	1455.4	-0.01097	0.9912	18
(Intercept)	7.21E-16	0.40825	1.77E-15	1.0000	19
Ia.CatNonsig	0.143100844	0.48833	0.293044	0.7695	19
(Intercept)	-0.223143551	0.3873	-0.57615	0.5645	20

Ia.CatNonsig	0.669430654	0.50249	1.332217	0.1828	20
(Intercept)	0.074107972	0.38516	0.192406	0.8474	21
Ia.CatNonsig	0.331357136	0.51711	0.640791	0.5217	21
(Intercept)	0.194156014	0.3609	0.537971	0.5906	22
Ia.CatNonsig	0.722134717	0.60298	1.197609	0.2311	22
(Intercept)	0.223143551	0.3873	0.576154	0.5645	23
Ia.CatNonsig	0.652325186	0.65828	0.990953	0.3217	23
(Intercept)	0.36772478	0.43363	0.848017	0.3964	24
Ia.CatNonsig	0.730887509	0.9245	0.790576	0.4292	24
(Intercept)	0.606135804	0.50752	1.194311	0.2324	25
Ia.CatNonsig	-0.200670695	1.04447	-0.19213	0.8476	25

Table S3.

Coefficients of SADIE clustering index vi of ash tree in local GLM models.

Explanatory	Estimate	Std. Error	z value	Pr(>|z|)	Plot radius (m)
(Intercept)	-0.43193	0.137684	-3.13712	0.0017	17
vi.CatPatch	-1.87065	1.057595	-1.76878	0.0769	17
(Intercept)	-0.44183	0.135107	-3.27024	0.0011	18
vi.CatPatch	-1.34993	1.08854	-1.24013	0.2149	18
(Intercept)	-0.41616	0.133585	-3.11533	0.0018	19
vi.CatPatch	-15.1499	727.6988	-0.02082	0.9834	19
(Intercept)	-0.44183	0.135107	-3.27024	0.0011	20
vi.CatPatch	-0.94446	0.802031	-1.17759	0.2390	20
(Intercept)	-16.5661	2399.545	-0.0069	0.9945	21
vi.CatRandom	16.1606	2399.545	0.006735	0.9946	21
vi.CatPatch	-1.87E-07	2529.342	-7.38E-11	1.0000	21
(Intercept)	-14.5661	882.7434	-0.0165	0.9868	22
vi.CatRandom	14.13414	882.7434	0.016012	0.9872	22
vi.CatPatch	12.08116	882.744	0.013686	0.9891	22
(Intercept)	-0.37194	0.136253	-2.7298	0.0063	23
vi.CatPatch	-1.57397	0.768107	-2.04915	0.0404	23
(Intercept)	-0.436	0.138372	-3.15095	0.0016	24
vi.CatPatch	-0.32614	0.478195	-0.68202	0.4952	24
(Intercept)	-14.5661	882.7434	-0.0165	0.9868	25
vi.CatRandom	14.05271	882.7434	0.015919	0.9873	25
vi.CatPatch	14.34292	882.7435	0.016248	0.9870	25

Table S4.

Coefficients of SADIE clustering index vi of DBH in local GLM models.

Explanatory	Estimate	Std. Error	z value	Pr(>|z|)	Plot radius (m)
(Intercept)	-2.19722	1.054006	-2.08464	0.0371	6
vi.CatRandom	1.879984	1.06488	1.765441	0.0775	6
vi.CatPatch	2.197225	1.763782	1.245746	0.2129	6
(Intercept)	-1.50408	0.781736	-1.92402	0.0544	7
vi.CatRandom	1.19042	0.796169	1.495186	0.1349	7
vi.CatPatch	0.81093	1.452966	0.558121	0.5768	7
(Intercept)	-1.60944	0.774597	-2.07778	0.0377	8
vi.CatRandom	1.325186	0.789942	1.677575	0.0934	8
vi.CatPatch	0.510826	1.390443	0.367383	0.7133	8
(Intercept)	-1.94591	1.069045	-1.82023	0.0687	9
vi.CatRandom	1.750166	1.080501	1.619773	0.1053	9
vi.CatPatch	-13.6202	1029.122	-0.01323	0.9894	9
(Intercept)	-2.63906	1.035095	-2.54958	0.0108	10
vi.CatRandom	2.497979	1.048681	2.382021	**0.0172**	10
vi.CatPatch	1.94591	1.603565	1.21349	0.2249	10
(Intercept)	-2.70805	1.032796	-2.62206	0.0087	11
vi.CatRandom	2.693451	1.046836	2.572945	**0.0101**	11
vi.CatPatch	-12.858	840.2748	-0.0153	0.9878	11
(Intercept)	-2.25129	0.743292	-3.02881	0.0025	12
vi.CatRandom	2.267821	0.765208	2.963665	**0.0030**	12
vi.CatPatch	1.845827	1.177207	1.567971	0.1169	12
(Intercept)	-2.89037	1.027368	-2.81338	0.0049	13
vi.CatRandom	2.961831	1.044627	2.835301	**0.0046**	13
vi.CatPatch	3.178054	1.280163	2.482538	**0.0130**	13
(Intercept)	-2.48491	1.040832	-2.38742	0.0170	14
vi.CatRandom	2.542065	1.058989	2.400463	**0.0164**	14
vi.CatPatch	2.079442	1.384437	1.502013	0.1331	14
(Intercept)	-1.20397	0.65828	-1.82897	0.0674	15
vi.CatRandom	1.248425	0.69123	1.806091	0.0709	15
vi.CatPatch	2.302585	1.048808	2.195431	**0.0281**	15
(Intercept)	-0.77319	0.493548	-1.5666	0.1172	16
vi.CatRandom	0.931414	0.544565	1.710381	0.0872	16
vi.CatPatch	1.689481	0.971385	1.739249	0.0820	16
(Intercept)	-0.33647	0.585518	-0.57466	0.5655	17
vi.CatRandom	0.518794	0.628659	0.825239	0.4092	17
vi.CatPatch	0.336472	1.004738	0.334886	0.7377	17
(Intercept)	-0.91629	0.591608	-1.54882	0.1214	18
vi.CatRandom	1.178655	0.639511	1.843058	0.0653	18
vi.CatPatch	1.427116	0.939858	1.518438	0.1289	18
(Intercept)	-1.38629	0.790569	-1.75354	0.0795	19
vi.CatRandom	1.645806	0.831028	1.980444	**0.0477**	19
vi.CatPatch	1.89712	1.076259	1.762699	0.0780	19
(Intercept)	-1.38629	0.645497	-2.14764	0.0317	20
vi.CatRandom	1.936341	0.722354	2.6806	**0.0073**	20

vi.CatPatch	2.079442	0.889756	2.337091	**0.0194**	20
(Intercept)	-1.60944	0.774597	-2.07778	0.0377	21
vi.CatRandom	2.189256	0.843462	2.595559	**0.0094**	21
vi.CatPatch	2.590267	1.028753	2.517871	**0.0118**	21
(Intercept)	-1.60944	0.774597	-2.07778	0.0377	22
vi.CatRandom	2.574519	0.878987	2.92896	**0.0034**	22
vi.CatPatch	3.113515	1.100505	2.82917	**0.0047**	22
(Intercept)	-1.38629	0.790569	-1.75354	0.0795	23
vi.CatRandom	2.197225	0.897527	2.448087	**0.0144**	23
vi.CatPatch	3.332205	1.329604	2.506162	**0.0122**	23
(Intercept)	-1.79176	1.080123	-1.65885	0.0971	24
vi.CatRandom	2.821379	1.199206	2.352706	**0.0186**	24
vi.CatPatch	20.35783	3261.319	0.006242	0.9950	24
(Intercept)	-1.38629	1.118034	-1.23994	0.2150	25
vi.CatRandom	2.079442	1.274755	1.631248	0.1028	25
vi.CatPatch	19.95236	2917.013	0.00684	0.9945	25

Table S5.

Coefficients of SADIE aggregation index Ia of pooled nonhost tree species in local GLM models.

Explanatory	Estimate	Std. Error	z value	Pr(>|z|)	Plot radius (m)
(Intercept)	15.56606831	1029.1215	0.015125589	0.9879	6
Ia.CatNonsig	-15.93560141	1029.1215	-0.015484665	0.9876	6
Ia.CatUniform	-6.33E-08	1782.4907	-3.55E-11	1.0000	6
(Intercept)	1.386294361	1.118034	1.239939371	0.2150	7
Ia.CatNonsig	-1.779020443	1.1274774	-1.577876842	0.1146	7
Ia.CatUniform	-16.95236261	1029.1221	-0.016472645	0.9869	7
(Intercept)	0.693147181	0.8660254	0.800377423	0.4235	8
Ia.CatNonsig	-1.084818967	0.8791802	-1.233898262	0.2172	8
Ia.CatUniform	-16.25921543	1029.1218	-0.015799116	0.9874	8
(Intercept)	1.71E-14	1	1.71E-14	1.0000	9
Ia.CatNonsig	-0.284251537	1.0119328	-0.28089963	0.7788	9
Ia.CatUniform	14.56606778	882.74394	0.016500898	0.9868	9
(Intercept)	1.098612289	1.1546995	0.95142697	0.3414	10
Ia.CatNonsig	-1.455287233	1.1663255	-1.247753939	0.2121	10
Ia.CatUniform	2.90E-15	1.6329918	1.78E-15	1.0000	10
(Intercept)	-2.81E-14	1.4142136	-1.99E-14	1.0000	11
Ia.CatNonsig	-0.223143551	1.4235416	-0.156752396	0.8754	11
Ia.CatUniform	-14.56606778	882.74451	-0.016500887	0.9868	11
(Intercept)	-16.56606845	1199.7724	-0.013807676	0.9890	12
Ia.CatNonsig	16.3682427	1199.7724	0.01364279	0.9891	12
Ia.CatUniform	33.13213687	2682.7726	0.012349961	0.9901	12
(Intercept)	-1.386294361	1.118034	-1.239939371	0.2150	13
Ia.CatNonsig	1.234488348	1.1315821	1.090940194	0.2753	13
Ia.CatUniform	15.95236214	882.74408	0.018071333	0.9856	13
(Intercept)	-0.693147181	1.2247446	-0.565952407	0.5714	14
Ia.CatNonsig	0.557345639	1.2385695	0.449991431	0.6527	14
Ia.CatUniform	0.693147181	1.8708285	0.370502782	0.7110	14
(Intercept)	1.098612289	1.1547005	0.951426151	0.3414	15
Ia.CatNonsig	-1.136352617	1.1709324	-0.970468187	0.3318	15
Ia.CatUniform	-16.66468054	1455.398	-0.011450257	0.9909	15
(Intercept)	-0.693147181	1.2247446	-0.565952407	0.5714	16
Ia.CatNonsig	0.753771802	1.2411449	0.607319751	0.5436	16
(Intercept)	-1.098612289	1.1546995	-0.95142697	0.3414	17
Ia.CatNonsig	1.252762968	1.1736902	1.067371059	0.2858	17
(Intercept)	-15.56606825	1455.3975	-0.010695407	0.9915	18
Ia.CatNonsig	15.69959965	1455.3975	0.010787156	0.9914	18
(Intercept)	4.38E-15	1	4.38E-15	1.0000	19
Ia.CatNonsig	0.133531393	1.0264363	0.130092238	0.8965	19
Ia.CatUniform	-15.56606825	1455.3979	-0.010695404	0.9915	19

(Intercept)	-17.56606849	1769.2576	-0.009928497	0.9921	20
Ia.CatNonsig	17.91888987	1769.2577	0.010127914	0.9919	20
(Intercept)	-1.098612289	1.1546995	-0.95142697	0.3414	21
Ia.CatNonsig	1.446918983	1.1850778	1.220948485	0.2221	21
(Intercept)	4.06E-15	1	4.06E-15	1.0000	22
Ia.CatNonsig	0.510825624	1.0434984	0.489531781	0.6245	22
(Intercept)	-0.405465108	0.9128709	-0.444164774	0.6569	23
Ia.CatNonsig	0.985283603	0.9719886	1.013678124	0.3107	23
(Intercept)	-2.53E-15	1.4142136	-1.79E-15	1.0000	24
Ia.CatNonsig	0.693147181	1.4719601	0.47090078	0.6377	24
Ia.CatUniform	-16.56606842	2399.5451	-0.006903837	0.9945	24
(Intercept)	1.098612289	1.1546995	0.95142697	0.3414	25
Ia.CatNonsig	-0.646627165	1.2518376	-0.516542392	0.6055	25

Data S1.

Global frequency table for vi index of ash tree

	Gap	Random	Patch
No	9	105	34
Yes	9	58	26

Data S2.

Global frequency table for vi index of the DBH of ash tree

	Gap	Random	Patch
No	49	83	16
Yes	5	50	38

Data S3.
Local frequency table for Ia index of ash tree

```
$`1`

     Aggregation Nonsig Uniform
  No           0    148       0
  Yes          0     93       0

$`2`

     Aggregation Nonsig Uniform
  No           0    146       0
  Yes          0     93       0

$`3`

     Aggregation Nonsig Uniform
  No           0    144       0
  Yes          0     93       0

$`4`

     Aggregation Nonsig Uniform
  No           0    139       0
  Yes          0     91       0

$`5`

     Aggregation Nonsig Uniform
  No           1    129       0
  Yes          0     91       0

$`6`

     Aggregation Nonsig Uniform
  No           2    120       1
  Yes          1     87       0

$`7`

     Aggregation Nonsig Uniform
  No           0    120       0
  Yes          1     82       0

$`8`
```

```
    Aggregation Nonsig Uniform
  No          1    111       0
  Yes         2     75       0

$`9`

    Aggregation Nonsig Uniform
  No          5     94       0
  Yes         0     76       0

$`10`

    Aggregation Nonsig Uniform
  No          5     87       0
  Yes         1     67       1

$`11`

    Aggregation Nonsig Uniform
  No          1     85       1
  Yes         1     67       1

$`12`

    Aggregation Nonsig Uniform
  No          2     79       1
  Yes         1     64       0

$`13`

    Aggregation Nonsig Uniform
  No          0     74       1
  Yes         0     63       0

$`14`

    Aggregation Nonsig Uniform
  No          2     62       2
  Yes         2     54       1

$`15`

    Aggregation Nonsig Uniform
  No          2     52       2
  Yes         3     52       0

$`16`
```

```
    Aggregation Nonsig Uniform
No            2     47       1
Yes           3     49       0

$`17`

    Aggregation Nonsig Uniform
No            1     42       2
Yes           4     46       0

$`18`

    Aggregation Nonsig Uniform
No            1     42       0
Yes           3     45       0

$`19`

    Aggregation Nonsig Uniform
No            2     36       0
Yes           1     40       1

$`20`

    Aggregation Nonsig Uniform
No            1     30       0
Yes           0     37       0

$`21`

    Aggregation Nonsig Uniform
No            3     24       0
Yes           0     35       0

$`22`

    Aggregation Nonsig Uniform
No            4     16       0
Yes           1     31       0

$`23`

    Aggregation Nonsig Uniform
No            2     15       0
Yes           2     25       0
```

```
$`24`

     Aggregation Nonsig Uniform
  No             1     10       0
  Yes            0     19       0

$`25`

     Aggregation Nonsig Uniform
  No             1      7       0
  Yes            0     14       0
```

Data S4.
Local frequency table for Ia index of DBH of ash tree

$`1`

	Aggregation	Nonsig	Uniform
No	4	23	0
Yes	0	8	0

$`2`

	Aggregation	Nonsig	Uniform
No	7	50	0
Yes	0	17	0

$`3`

	Aggregation	Nonsig	Uniform
No	8	74	0
Yes	0	36	1

$`4`

	Aggregation	Nonsig	Uniform
No	7	104	0
Yes	0	60	0

$`5`

	Aggregation	Nonsig	Uniform
No	12	100	1
Yes	2	68	0

$`6`

	Aggregation	Nonsig	Uniform
No	11	98	4
Yes	3	74	0

$`7`

	Aggregation	Nonsig	Uniform
No	8	104	3
Yes	4	73	2

$`8`

```
     Aggregation Nonsig Uniform
  No             9     97       4
  Yes            3     71       2

$`9`

     Aggregation Nonsig Uniform
  No             5     93       1
  Yes            2     69       4

$`10`

     Aggregation Nonsig Uniform
  No             4     86       2
  Yes            0     65       3

$`11`

     Aggregation Nonsig Uniform
  No             4     83       0
  Yes            2     65       2

$`12`

     Aggregation Nonsig Uniform
  No             7     74       1
  Yes            1     62       2

$`13`

     Aggregation Nonsig Uniform
  No             4     71       0
  Yes            3     60       0

$`14`

     Aggregation Nonsig Uniform
  No             5     61       0
  Yes            7     50       0

$`15`

     Aggregation Nonsig Uniform
  No             6     49       1
  Yes           10     45       0

$`16`
```

```
    Aggregation Nonsig Uniform
  No            9     40       1
  Yes          11     40       1

$`17`

    Aggregation Nonsig Uniform
  No            8     37       0
  Yes          11     39       0

$`18`

    Aggregation Nonsig Uniform
  No            8     34       1
  Yes          12     36       0

$`19`

    Aggregation Nonsig Uniform
  No           12     26       0
  Yes          12     30       0

$`20`

    Aggregation Nonsig Uniform
  No           15     16       0
  Yes          12     25       0

$`21`

    Aggregation Nonsig Uniform
  No           13     14       0
  Yes          14     21       0

$`22`

    Aggregation Nonsig Uniform
  No           14      6       0
  Yes          17     15       0

$`23`

    Aggregation Nonsig Uniform
  No           12      5       0
  Yes          15     12       0
```

```
$`24`

     Aggregation Nonsig Uniform
  No           9      2       0
  Yes         13      6       0

$`25`

     Aggregation Nonsig Uniform
  No           6      2       0
  Yes         11      3       0
```

Data S5.
Local frequency table for vi index of ash tree

```
$`1`

      Gap Random Patch
  No    0     148     0
  Yes   0      93     0

$`2`

      Gap Random Patch
  No    0     146     0
  Yes   0      93     0

$`3`

      Gap Random Patch
  No    0     144     0
  Yes   0      93     0

$`4`

      Gap Random Patch
  No    0     139     0
  Yes   0      91     0

$`5`

      Gap Random Patch
  No    0     130     0
  Yes   0      90     0

$`6`

      Gap Random Patch
  No    0     122     0
  Yes   0      86     0

$`7`

      Gap Random Patch
  No    0     118     0
  Yes   0      82     0

$`8`

      Gap Random Patch
```

```
  No    0     111     1
  Yes   0      76     0

$`9`

      Gap Random Patch
  No    0      95     0
  Yes   0      74     0

$`10`

      Gap Random Patch
  No    0      88     0
  Yes   0      66     0

$`11`

      Gap Random Patch
  No    0      79     0
  Yes   0      67     0

$`12`

      Gap Random Patch
  No    0      81     0
  Yes   0      60     0

$`13`

      Gap Random Patch
  No    0      74     0
  Yes   0      63     0

$`14`

      Gap Random Patch
  No    0      66     0
  Yes   0      56     0

$`15`

      Gap Random Patch
  No    0      56     0
  Yes   0      51     1

$`16`
```

```
      Gap Random Patch
  No    0     50     0
  Yes   0     51     0

$`17`

      Gap Random Patch
  No    0     39     5
  Yes   0     45     1

$`18`

      Gap Random Patch
  No    0     41     2
  Yes   0     47     0

$`19`

      Gap Random Patch
  No    0     35     2
  Yes   0     42     0

$`20`

      Gap Random Patch
  No    0     29     2
  Yes   0     36     0

$`21`

      Gap Random Patch
  No    0     22     5
  Yes   0     34     0

$`22`

      Gap Random Patch
  No    0     17     3
  Yes   0     30     0

$`23`

      Gap Random Patch
  No    0     16     1
  Yes   0     27     0

$`24`
```

```
     Gap Random Patch
  No    0     10     1
  Yes   0     18     1

$`25`

     Gap Random Patch
  No    0      8     0
  Yes   0     13     1
```

Data S6.

Frequency table for vi index of DBH of ash tree

```
$`1`

      Gap Random Patch
  No    0     27     0
  Yes   0      8     0

$`2`

      Gap Random Patch
  No    0     55     1
  Yes   0     17     0

$`3`

      Gap Random Patch
  No    0     80     2
  Yes   0     37     0

$`4`

      Gap Random Patch
  No    2    107     2
  Yes   0     58     2

$`5`

      Gap Random Patch
  No    7    105     1
  Yes   0     68     2

$`6`

      Gap Random Patch
  No    9    103     1
  Yes   1     75     1

$`7`

      Gap Random Patch
  No    9    104     2
  Yes   2     76     1

$`8`

      Gap Random Patch
```

```
  No   10       97      3
  Yes   2       73      1

$`9`

      Gap Random Patch
  No    7       90      2
  Yes   1       74      0

$`10`

      Gap Random Patch
  No   14       76      2
  Yes   1       66      1

$`11`

      Gap Random Patch
  No   15       69      3
  Yes   1       68      0

$`12`

      Gap Random Patch
  No   19       60      3
  Yes   2       61      2

$`13`

      Gap Random Patch
  No   18       54      3
  Yes   1       58      4

$`14`

      Gap Random Patch
  No   12       51      3
  Yes   1       54      2

$`15`

      Gap Random Patch
  No   10       44      2
  Yes   3       46      6

$`16`
```

```
      Gap Random Patch
  No   13     35     2
  Yes   6     41     5

$`17`

      Gap Random Patch
  No    7     35     3
  Yes   5     42     3

$`18`

      Gap Random Patch
  No   10     30     3
  Yes   4     39     5

$`19`

      Gap Random Patch
  No    8     27     3
  Yes   2     35     5

$`20`

      Gap Random Patch
  No   12     15     4
  Yes   3     26     8

$`21`

      Gap Random Patch
  No   10     14     3
  Yes   2     25     8

$`22`

      Gap Random Patch
  No   10      8     2
  Yes   2     21     9

$`23`

      Gap Random Patch
  No    8      8     1
  Yes   2     18     7

$`24`
```

```
     Gap Random Patch
  No    6      5     0
  Yes   1     14     4

$`25`

     Gap Random Patch
  No    4      4     0
  Yes   1      8     5
```

Data S7.

Frequency table for Ia index of pooled nonhost tree species

```
$`1`

     Aggregation Nonsig Uniform
  No           0    148       0
  Yes          0     93       0

$`2`

     Aggregation Nonsig Uniform
  No           0    146       0
  Yes          0     93       0

$`3`

     Aggregation Nonsig Uniform
  No           0    144       0
  Yes          0     93       0

$`4`

     Aggregation Nonsig Uniform
  No           0    139       0
  Yes          0     91       0

$`5`

     Aggregation Nonsig Uniform
  No           2    128       0
  Yes          0     90       1

$`6`

     Aggregation Nonsig Uniform
  No           0    123       0
  Yes          2     85       1

$`7`

     Aggregation Nonsig Uniform
  No           1    117       2
  Yes          4     79       0

$`8`

     Aggregation Nonsig Uniform
```

```
  No             2    108       2
  Yes            4     73       0

$`9`

      Aggregation Nonsig Uniform
  No             2     97       0
  Yes            2     73       1

$`10`

      Aggregation Nonsig Uniform
  No             1     90       1
  Yes            3     63       3

$`11`

      Aggregation Nonsig Uniform
  No             1     85       1
  Yes            1     68       0

$`12`

      Aggregation Nonsig Uniform
  No             4     78       0
  Yes            0     64       1

$`13`

      Aggregation Nonsig Uniform
  No             4     71       0
  Yes            1     61       1

$`14`

      Aggregation Nonsig Uniform
  No             2     63       1
  Yes            1     55       1

$`15`

      Aggregation Nonsig Uniform
  No             1     54       1
  Yes            3     52       0

$`16`
```

```
    Aggregation Nonsig Uniform
No            2     48       0
Yes           1     51       0

$`17`

    Aggregation Nonsig Uniform
No            3     42       0
Yes           1     49       0

$`18`

    Aggregation Nonsig Uniform
No            1     42       0
Yes           0     48       0

$`19`

    Aggregation Nonsig Uniform
No            2     35       1
Yes           2     40       0

$`20`

    Aggregation Nonsig Uniform
No            5     26       0
Yes           0     37       0

$`21`

    Aggregation Nonsig Uniform
No            3     24       0
Yes           1     34       0

$`22`

    Aggregation Nonsig Uniform
No            2     18       0
Yes           2     30       0

$`23`

    Aggregation Nonsig Uniform
No            3     14       0
Yes           2     25       0

$`24`
```

```
     Aggregation Nonsig Uniform
  No            1      9       1
  Yes           1     18       0

$`25`

     Aggregation Nonsig Uniform
  No            1      7       0
  Yes           3     11       0
```
